# Statistical Analysis Is Vital at *eNeuro*

**DOI:** 10.1523/ENEURO.0082-24.2024

**Published:** 2024-03-12

**Authors:** Christophe Bernard

**Affiliations:** Editor in Chief, *eNeuro*

In our continuous drive to increase the standards of statistical rigor in neuroscience, we, at *eNeuro*, have launched a series of initiatives aimed at refining how statistics are employed, reported, and interpreted in neuroscience (Bernard, 2019, 2021; Calin-Jageman and Cumming, 2019). The drive toward this enhancement stems, in part, from the ongoing challenge of reproducibility—a cornerstone of scientific integrity that, unfortunately, has been undermined by gaps in statistical literacy (Bernard, 2023).

Statistics, a discipline with its unique complexities and nuances, extends far beyond mere number-crunching. My personal journey from grasping basic statistical concepts to applying sophisticated analytical methods in my research has been both enlightening and humbling. Yet I often encountered statistical methodologies with which I was unfamiliar. This realization is already a problem when reading a paper, but it becomes a major hurdle when reviewing a paper. Historically, the absence of a structured process for addressing these gaps in scientific journals meant that potential issues could slip through the cracks, compromising the rigor of published research.

To address this, we have introduced a mandatory checkpoint for our reviewers at *eNeuro*, focusing specifically on the appropriateness of statistical analyses within submissions. Reviewers are prompted to assess whether the statistical methodologies are accurately applied, offering responses that range from affirmative to uncertain. Uncertainties trigger an invaluable process, requesting the expertise of expert statisticians to ensure the soundness of the analysis, with a single final goal: providing help to both authors and reviewers.

To support these existing initiatives, *eNeuro* added a new Statistics Editor position to the Advisory Board in 2023, and I am delighted that Robert Calin-Jageman agreed to serve in this role. Many of you already read his editorials at *eNeuro* or his textbook (Cumming and Calin-Jageman, 2016). Bob will help us in solving statistical issues, either directly or by contacting specialists in the various statistical fields.

At the heart of *eNeuro*’s mission is a commitment to education. We believe that empowering researchers with a thorough understanding of statistical tools is fundamental to advancing neuroscience. Our dedication to this cause is evident in the numerous initiatives we have launched, aimed at fostering a culture of continuous learning and improvement. In this spirit, I am pleased to collaborate with Bob on a new series of commentaries focused not only on statistical analysis but also on ways to improve our neuroscience (Calin-Jageman, 2024). This initiative, which will include webinars, is designed to enhance our collective proficiency in applying statistical principles, thereby elevating the quality of our research and peer evaluations.

In closing, I invite you all to join us in this exciting phase of growth and learning. As always, if you wish to contribute to such initiatives, do not hesitate to contact me directly.
